# Bronchioalveolar stem cells derived from mouse-induced pluripotent stem cells promote airway epithelium regeneration

**DOI:** 10.1186/s13287-020-01946-7

**Published:** 2020-10-02

**Authors:** Naoya Kawakita, Hiroaki Toba, Keiko Miyoshi, Shinichi Sakamoto, Daisuke Matsumoto, Mika Takashima, Mariko Aoyama, Seiya Inoue, Masami Morimoto, Takeshi Nishino, Hiromitsu Takizawa, Akira Tangoku

**Affiliations:** 1 Department of Thoracic and Endocrine Surgery and Oncology, Institute of Biomedical Sciences, The University of Tokushima Graduate School, 3-18-15, Kuramoto-cho, Tokushima, 770-8503 Japan; 2 Department of Molecular Biology, Institute of Biomedical Sciences, Tokushima University Graduate School, Tokushima, Japan; 3 Department of Breast Surgery, Japanese Red Cross Kyoto Daiichi Hospital, Kyoto, Japan

**Keywords:** Induced pluripotent stem cells, Stem cell transplantation, Progenitor cells, Tissue regeneration

## Abstract

**Background:**

Bronchioalveolar stem cells (BASCs) located at the bronchioalveolar-duct junction (BADJ) are stem cells residing in alveoli and terminal bronchioles that can self-renew and differentiate into alveolar type (AT)-1 cells, AT-2 cells, club cells, and ciliated cells. Following terminal-bronchiole injury, BASCs increase in number and promote repair. However, whether BASCs can be differentiated from mouse-induced pluripotent stem cells (iPSCs) remains unreported, and the therapeutic potential of such cells is unclear. We therefore sought to differentiate BASCs from iPSCs and examine their potential for use in the treatment of epithelial injury in terminal bronchioles.

**Methods:**

BASCs were induced using a modified protocol for differentiating mouse iPSCs into AT-2 cells. Differentiated iPSCs were intratracheally transplanted into naphthalene-treated mice. The engraftment of BASCs into the BADJ and their subsequent ability to promote repair of injury to the airway epithelium were evaluated.

**Results:**

Flow cytometric analysis revealed that BASCs represented ~ 7% of the cells obtained. Additionally, ultrastructural analysis of these iPSC-derived BASCs via transmission electron microscopy showed that the cells containing secretory granules harboured microvilli, as well as small and immature lamellar body-like structures. When the differentiated iPSCs were intratracheally transplanted in naphthalene-induced airway epithelium injury, transplanted BASCs were found to be engrafted in the BADJ epithelium and alveolar spaces for 14 days after transplantation and to maintain the BASC phenotype. Notably, repair of the terminal-bronchiole epithelium was markedly promoted after transplantation of the differentiated iPSCs.

**Conclusions:**

Mouse iPSCs could be differentiated in vitro into cells that display a similar phenotype to BASCs. Given that the differentiated iPSCs promoted epithelial repair in the mouse model of naphthalene-induced airway epithelium injury, this method may serve as a basis for the development of treatments for terminal-bronchiole/alveolar-region disorders.

## Background

The lung features a complex internal structure that harbours multiple epithelial cell types, including bronchial and alveolar epithelial cells, as well as pulmonary endothelial cells. The lung epithelium plays specialised roles in respiration and host defence, and lung epithelial cells can be repaired following damage by infection, air pollutants, and various irritants. This repair of the lung and bronchiole epithelium is governed by stem cell populations present in distinct niches along the proximal–distal axis [1, 2]. Interaction between lung stem cells and their niche is critical for maintaining the balance between stem cells and differentiated cells, while an imbalance between these cell populations causes inappropriate repair and leads to the development of lung diseases, such as chronic obstructive pulmonary disease, idiopathic pulmonary fibrosis, and lung cancer [3].

In the terminal bronchioles of the mouse lung, the percentage of club cells among epithelial cells is known to be high (~ 90%) [4]. A widely recognised club cell-specific cytotoxicant is naphthalene; therefore, the mouse model of naphthalene-induced peripheral airway injury is frequently used in regeneration studies [5–12]. Following club cell depletion, the residual cells in the terminal bronchioles can complete the repair of the epithelium and regenerate the cells appropriate for this lung region [11–13]. In this process, the stem cells that renew the terminal-bronchiole epithelium by proliferating and differentiating into club cells are called bronchioalveolar stem cells (BASCs) [2, 6, 10, 14–24].

However, to date, no drug or air pollutant that specifically injures human club cells has been identified. In addition, human airway constituent cells differ from those in mice, and the proportion of club cells in the terminal bronchi of humans is rather low (10–40%) [4]. Nevertheless, even in humans, club cells not only contribute to host defence by producing anti-inflammatory club cell secretory protein (CCSP), but also represent an abundant progenitor cell that functions to maintain the health of distal airways via repair of epithelium after injury [10]. In particular, club cell injury is regarded as important during the development of bronchiolitis obliterans syndrome, which is a complication that can occur after lung transplantation and is highly associated with late death [25–27]. Hence, research to establish methods for repair following club cell injury in humans is becoming increasingly important.

BASCs that localise at the bronchioalveolar-duct junction (BADJ) and co-express markers of club cells and alveolar type (AT)-2 cells have recently been regarded as local stem cells used for lung repair. BASCs can self-renew and differentiate into club cells, AT-1 cells, AT-2 cells, and ciliated cells [22, 23, 28]. Notably, BASCs increase in number during the repair process in naphthalene-induced airway injury [6, 19]. Therefore, we hypothesised that BASC transplantation might promote repair in naphthalene-induced airway injury. BASCs can be isolated from adult mouse lungs using flow cytometry; however, an inherent challenge associated with this strategy is that the BASC population represents < 1% of the total lung cell population [14, 29].

Induced pluripotent stem cells (iPSCs), which are obtained by introducing Yamanaka factors (OCT4, SOX2, KLF4, cMYC: OSKM) into differentiated somatic cells, display self-renewal properties and pluripotency [30]. AT-2 cells or lung progenitor cells have been derived from human or mouse iPSCs and embryonic stem cells (ESCs) in several studies either through embryoid body (EB) formation or by using differentiation medium. Furthermore, administration of these iPSC-derived cells to mouse models of LPS-induced lung injury, bleomycin-induced lung injury, and hyperoxia-induced lung injury has been shown to reduce injury, promote repair, and improve lung function [31–47]. Therefore, iPSC-derived cells are considered to have therapeutic potential. However, no study thus far has reported the in vitro differentiation of iPSCs into BASCs, and the therapeutic potential of BASCs remains unclear. Here, we generated iPSC-derived BASCs and intratracheally transplanted these cells in a mouse model of naphthalene-induced airway injury to evaluate their therapeutic potential in the repair of damaged club cells.

## Methods

### Cell line and culture

A mouse iPSC line (iPS-MEF-Ng-492B-4) was purchased from the RIKEN Cell Bank (Tsukuba, Japan; http://www.brc.riken.go.jp/lab/cell) and maintained on a feeder layer of 3 × 10^4^ cells/cm^2^ mitomycin C-inactivated mouse SNL76/7 cells (European Cell Culture Collection, Porton Down, UK, EC07032801); mitomycin C was purchased from Kyowa Kirin Co., Ltd. (Tokyo, Japan). The cells were cultured on 0.1% gelatin-coated tissue-culture dishes, in iPS medium containing KnockOut™ Dulbecco’s modified Eagle’s medium (DMEM; Gibco, Carlsbad, CA) supplemented with 15% KnockOut™ Serum Replacement (Gibco), 0.1 mM nonessential amino acids (Gibco), 0.1 mM 2-mercaptoethanol (Gibco), 1× GlutaMAX™-I (Gibco), and 1000 U/mL murine Leukaemia Inhibitory Factor (Wako Pure Chemical Industries, Osaka, Japan) at 37 °C in a humidified atmosphere of 5% CO_2_. This iPSC line expresses green fluorescent protein (GFP) under the control of the *Nanog* promoter.

### iPSC differentiation

**Fig. 1:**
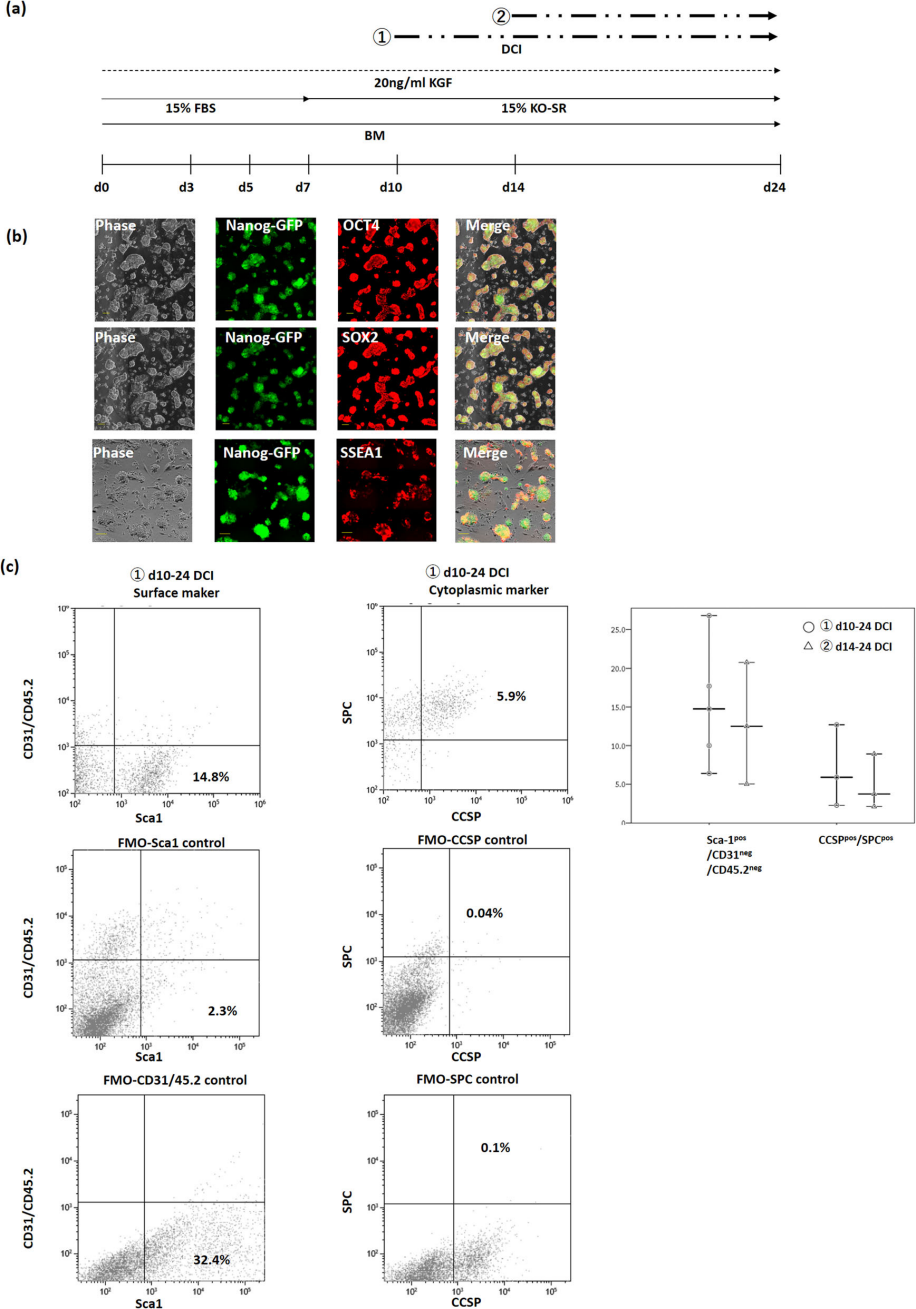
Differentiation of iPSCs into BASCs. **a** Schema of iPSC differentiation procedure: iPSCs were differentiated for 24 days through hanging-drop-based formation of embryoid bodies (EBs); the BM was supplemented from 0 to 24 days with 20 ng/mL keratinocyte growth factor (KGF) and DCI (① d10–d24 or ② d14–d24). EBs were induced using the hanging-drop method for the first 3 days, and the obtained EBs were transferred at 3 days to super-low-adherent culture dishes and then at 5 days to adherent culture dishes. Cells were cultured until 24 days in the medium. **b** Pluripotency of undifferentiated iPSCs (0 days) at passage 25. Immunofluorescence labelling of mouse iPSCs for the stem cell markers OCT4, SOX2, and SSEA-1. The *GFP* gene was knocked-in under the *Nanog* promoter, which allowed detection of GFP (green) in undifferentiated cells. Scale bar = 100 μm. **c** Flow cytometry analysis for BASC identification. Comparison of protocols ① d10–d24 DCI and ② d14–d24 DCI revealed that BASC differentiation efficiency did not differ significantly between the protocols (*P* > 0.05, Student’s *t* test); horizontal line inside indicates median and whiskers indicate min to max values. DCI, 10 nM dexamethasone plus 0.1 mM 8-bromoadenosine 3′5′-cyclic monophosphate sodium salt and 0.1 mM 3-isobutyl-1-methylxanthine; iPSCs, induced pluripotent stem cells; BASCs, bronchioalveolar stem cells; GFP, green fluorescent protein

To induce differentiation of mouse iPSCs into BASCs, a previously reported method for inducing AT-2 cells was used [39]. Briefly, mouse iPSCs were harvested using TrypLE™ Express Enzyme solution (Gibco) and incubated for 1 h on 0.1% gelatin-coated dishes. During this procedure, a majority of the feeder SNL cells contained in the cell suspension reattached to the bottom of the dishes. The supernatant containing the iPSCs was collected and the cell concentration was adjusted to 3 × 10^4^/mL using the differentiation medium described below. To initiate EB formation, the hanging-drop method was used; 20-μL drops of the cell suspension in differentiation medium containing ~ 600 mixed cells were placed on the lid of bacteriological Petri dishes and incubated for 3 days, and EBs were then transferred to 96-well cell-repellent plates and cultivated for 2 days. After 5 days, EBs were plated onto 6-well culture dishes (10 EBs/well) coated with 0.1% gelatin and cultivated until 24 days. The basal differentiation medium (BM) was composed of Iscove’s modified Dulbecco’s medium (Gibco), 0.2 mM L-glutamine (Gibco), 0.1 mM 2-mercaptoethanol, and 0.1 mM nonessential amino acids (Gibco). The BM was supplemented from 0 to 7 days with 15% foetal bovine serum (Gibco) and from 7 to 24 days with 15% KnockOut™ Serum Replacement. The BM was further supplemented with the following growth factors: 20 ng/mL recombinant human keratinocyte growth factor (KGF; ProteinTech Inc., Tokyo, Japan) (from 0 to 24 days) and DCI (treatment protocol: ① d10–d24 or ② d14–d24). DCI is a three-factor combination of 10 nM dexamethasone (Sigma-Aldrich, St. Louis, MO) plus 0.1 mM 8-bromoadenosine 3′5′-cyclic monophosphate sodium salt (Sigma-Aldrich) and 0.1 mM 3-isobutyl-1-methylxanthine (Sigma-Aldrich). The medium was replaced every 2–3 days. The differentiation procedure is depicted schematically in Fig. 1a.

### Animal care

Animals were housed in Micro-Isolator cages on a layer of wood shavings at a temperature of 22 °C, under a fixed 12/12-h light/dark cycle. All studies were performed in accordance with the guidelines established by the Tokushima University committee on animal care and use. All experimental protocols were reviewed and approved by the IACUC of the Tokushima University, Japan (T2019-8).

### Experimental design

Female C57/BL6 mice (8–12 weeks old) were used in all experiments. Mice in the corn oil group were intraperitoneally treated with corn oil (Sigma-Aldrich) at a dose of 10 mL/kg body weight and then sacrificed 5 days after the treatment (*n* = 4). Mice in the naphthalene-treatment group (NA group), control group, and iPS group were intraperitoneally treated with naphthalene (Sigma-Aldrich) dissolved in corn oil at a dose of 200 mg/kg body weight. On the day following the naphthalene treatment, mice in the control and iPS groups were anaesthetised using isoflurane inhalation. After the mice were fixed on an intubation stand (KN-1014; Natsume Seisakusho Co., Ltd., Tokyo, Japan), a 20-gauge plastic cannula was placed in the trachea through the glottis using a small animal laryngoscope (KN-1022; Natsume Seisakusho). The control group mice then received a single intratracheal injection of 50 μL of DMEM and the iPS group received a single intratracheal injection of differentiated iPSCs (1.0 × 10^6^ cells/mouse, in 50 μL of DMEM). The transplanted cells were labelled using a PKH26 Red Fluorescent Cell Linker Mini kit (Sigma-Aldrich), according to the manufacturer’s protocol. The mice in each group were sacrificed at two time points: 5 days and 15 days (*n* = 4–5/group, at each time point).

### Histological analysis

Differentiated iPSCs were dissociated into single-cell suspensions in PBS and centrifuged. The pellets were fixed with 10% formaldehyde for 1 h and embedded in paraffin. The paraffin-embedded specimens were cut at 5-μm thickness for haematoxylin and eosin (H&E) staining or immunofluorescence analysis.

After mice were sacrificed, the lungs from the corn oil group, NA group, and control group were harvested, and the left lung, which was used for histological studies, was fixed with 10% buffered formalin injected intratracheally; the remaining lung tissue was snap-frozen and stored at − 80 °C for RNA extraction. The left lung was cut in half along the major axis of the left main bronchus. Both sections were then embedded in paraffin and cut at 5-μm thickness. The sections were stained with H&E or used for immunofluorescence analysis. The left lungs from mice in the iPS group were fixed (overnight, in the dark) using a mixture of 4% paraformaldehyde and optimal cutting temperature (OCT) compound (4:1) administered intratracheally; subsequently, the lungs were cut in half along the major axis of the left main bronchus. One half was embedded in OCT compound and frozen, and the other was embedded in paraffin. The frozen specimens were cut at 10-μm thickness for immunofluorescence analysis, and the paraffin-embedded specimens were cut at 5-μm thickness for H&E staining or immunofluorescence analysis.

### Flow cytometry

To quantify BASCs among differentiated cells, flow cytometry was performed for (1) cytoplasmic markers and (2) surface markers. In the case of cytoplasmic markers, CCSP-positive and surfactant protein C (SPC)-positive (CCSP^pos^/SPC^pos^) cells were quantified. Briefly, differentiated cells were dissociated into single-cell suspensions in PBS, and then IntraPrep permeabilisation reagent (A07802; Beckman Coulter, Fullerton, CA) was used according to the manufacturer’s protocol. The cells were stained for 15 min on ice with anti-mouse CCSP antibody conjugated with Alexa Fluor® 488 (1:100, sc-365992 AF488; Santa Cruz Biotechnology, Santa Cruz, CA) and anti-mouse SPC antibody conjugated with Alexa Fluor® 647 (1:100, bs-10067R-A647; Bioss Inc., Boston, MA). Flow cytometry was performed using a BD FACSVerse Flow Cytometer running BD FACSuite Software (BD Biosciences, San Jose, CA) and Kaluza Analysis Software (Beckman Coulter). In the case of surface markers, Sca-1^pos^/CD45.2^neg^/CD31^neg^ cells were quantified according to previous reports [14, 29]. Single-cell suspensions of differentiated cells were stained for 15 min on ice with anti-mouse Sca-1 conjugated with FITC (1:100, #557405; BD Biosciences), anti-mouse CD45.2 conjugated with APC (1:100, #561875; BD Biosciences), and anti-mouse CD31 conjugated with APC (1:100, #561814; BD Biosciences). Flow cytometry was performed using a Cell Sorter LE-SH800S running SH800 software (SONY, Tokyo, Japan) and Kaluza Analysis Software.

### Transmission electron microscopy (TEM)

From differentiated iPSCs, the Sca-1^pos^/CD45.2^neg^/CD31^neg^ cell population was isolated using fluorescence-activated cell sorting (FACS). The sorted cells were fixed with 2% glutaraldehyde in 0.1 M phosphate buffer overnight at 4 °C, washed in 0.1 M phosphate buffer, and post-fixed with 2% osmium tetroxide in 0.1 M phosphate buffer for 2 h at 4 °C. Next, the cells were dehydrated using a graded ethanol series, incubated in propylene oxide for 20 min, and impregnated by incubation in propylene oxide/epoxy resin (2:1 v/v) for 2 h followed by propylene oxide/epoxy resin (1:1 v/v) overnight. On the following day, the samples were embedded in Epon812 (48 h at 60 °C). Ultrathin sections (80 nm) were cut using an ultramicrotome, and for contrasting, the sections were incubated with 2% uranyl acetate for 15 min and lead citrate for 5 min. Lastly, the sections were imaged using an H-700 transmission electron microscope (HITACHI, Tokyo, Japan) operating at 100 kV.

### Immunofluorescence

Undifferentiated iPSCs in 24-well plates were subjected to live staining with mouse anti-mouse SSEA-1 antibody conjugated with DyLight™ 550 (StainAlive™ SSEA-1 Antibody, 09-00959; Stemgent, San Diego, CA). The cells were fixed with 4% paraformaldehyde in PBS for 20 min and blocked with 10% goat serum (PCN5000; Invitrogen, Carlsbad, CA) in PBS containing 0.1% Triton X-100 for 1 h at room temperature. After overnight incubation at 4 °C with primary antibodies (1:100), rabbit anti-Sox2 (09-0024; Stemgent) or rabbit anti-Oct4 (09-0023; Stemgent), the wells were washed with PBS, and the cells were exposed to the secondary antibody, anti-rabbit IgG conjugated with Alexa Fluor® 594 (1:100, A-11037; Invitrogen), for 1 h at room temperature. Lastly, the wells were washed with PBS and the cells were counterstained with 4′,6-diamidino-2-phenylindole dihydrochloride (DAPI) (D3571; Invitrogen).

Paraffin-embedded sections were deparaffinised and antigens were retrieved using 2100 Antigen Retriever (Aptum Biologics Ltd.) containing 10 mM citric buffer (pH 6) for 2.5 h. The sections were blocked with 5% BSA/PBS for 2 h at room temperature and the following directly conjugated antibodies (1:100 in 5% BSA/PBS) were then used for labelling: anti-mouse CCSP antibody conjugated with Alexa Fluor® 488 (sc-365992 AF488; Santa Cruz Biotechnology) and anti-mouse SPC antibody conjugated with Alexa Fluor® 647 (bs-10067R-A647; Bioss Inc.). Mouse IgG1 conjugated with Alexa Fluor® 488 (#4878S; Cell Signaling Technology, Danvers, MA) and rabbit IgG conjugated with Alexa Fluor® 647 (#3452S; Cell Signaling Technology) were used as isotype controls. After incubation with the antibodies for 1 h at room temperature in a humidified chamber, the sections were washed four times in PBS/0.3% Triton X and mounted with ProLong™ Diamond Antifade Mountant containing DAPI (P36966; Invitrogen). Fluorescence images were captured using a fluorescence microscope (BZ-X800, Keyence; Osaka, Japan). CCSP-positive cells located within 200 μm of the BADJ and exhibiting a specific nuclear labelling profile with clear attachment to the basement membrane were counted as club cells, from a minimum of five BADJs [10].

### Statistical analysis

All analyses were conducted using SPSS software (version 24, IBM Corp., Armonk, NY). All statistical tests were two-sided, and *P* < 0.05 was considered significant. All continuous values are expressed as means ± standard deviation (SD). Student’s *t* test was used to compare two groups of continuous variables. For comparing continuous variables of three or more groups, one-way analysis of variance (ANOVA) followed by Tukey’s multiple-comparison test was used.

## Results

### Differentiation of iPSCs into BASCs

**Fig. 2:**
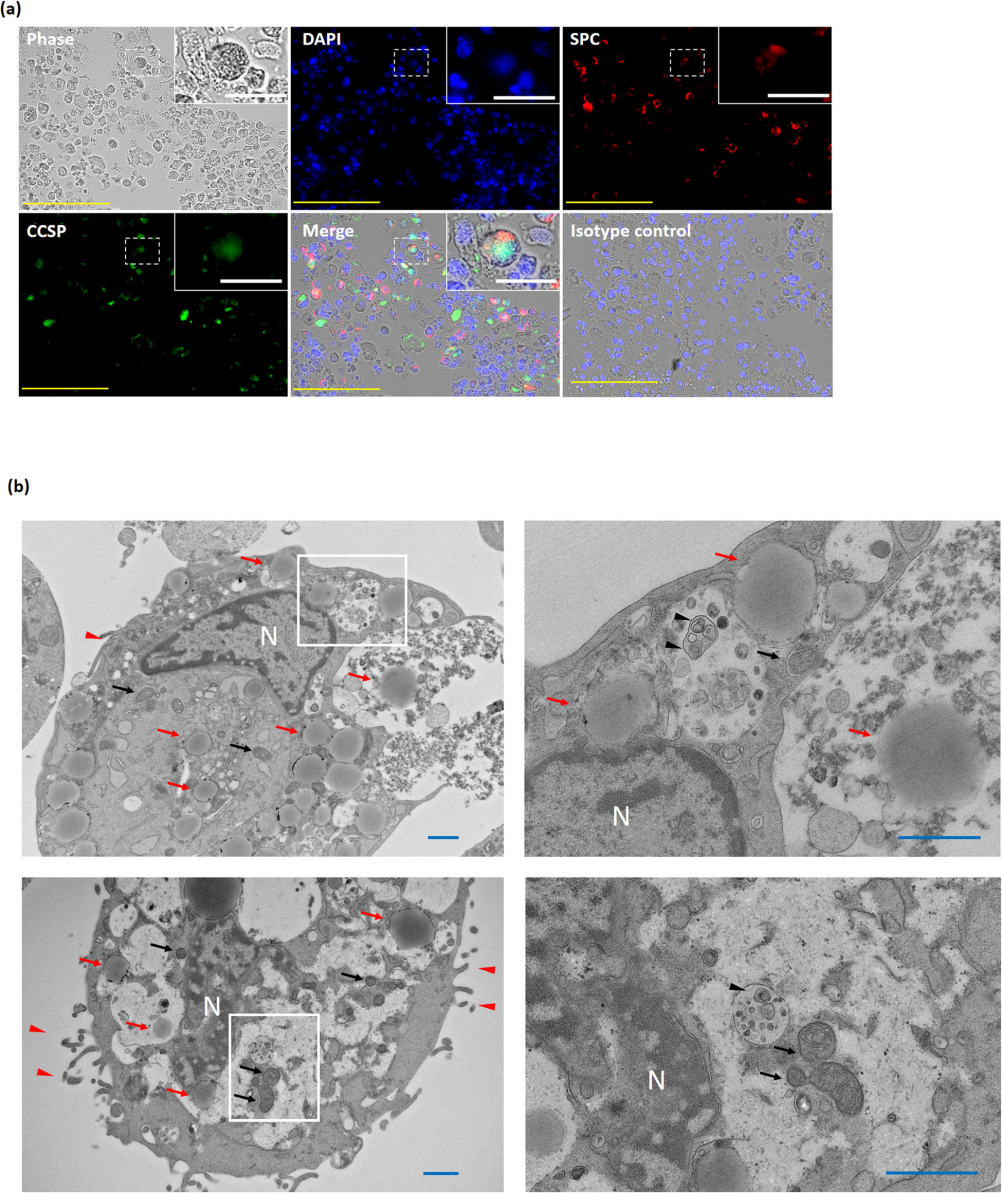
Identification of BASCs. Transmission electron micrographs of iPSC-derived BASCs. **a** Immunofluorescence labelling of iPSC-derived differentiated cells. Yellow scale bar = 100 μm; white scale bar = 20 μm. **b** A single BASC is shown containing microvilli (red arrowhead), immature lamellar body-like structures (black arrowhead), and secretory granules (red arrow); black arrow, mitochondria; N, nucleus. Scale bar = 1 μm. iPSCs, induced pluripotent stem cells; BASCs, bronchioalveolar stem cells; CCSP, club cell secretory protein; DAPI, 4′,6-diamidino-2-phenylindole dihydrochloride; SPC, surfactant protein C

Mouse iPSCs were maintained in culture and their undifferentiated status was monitored until passage 25. Immunostaining for the mouse pluripotency markers SSEA-1, NANOG, OCT4, and SOX2 revealed that the iPSCs were positive for all four markers (Fig. 1b), indicating that the iPSCs maintained their pluripotency. In this study, mouse iPSCs from passage 18 to passage 25 were used. The FACS analysis results at differentiation d24 showed that Sca-1^pos^/CD45.2^neg^/CD31^neg^ cells, which included BASCs, constituted 15.1 ± 7.8% and 12.8 ± 7.9% of the cells in ① d10–d24 DCI and ② d14–d24 DCI groups, respectively. Moreover, the CCSP^pos^/SPC^pos^ cell population, in which BASCs were further enriched, constituted 7.0 ± 5.3% and 4.9 ± 3.6% of the cells in ① d10–d24 DCI and ② d14–d24 DCI groups, respectively. The BASC ratio did not differ significantly between ① d10–d24 DCI and ② d14–d24 DCI groups (Fig. 1c). According to the labelling for both surface and intracellular markers, the ratio of BASCs in ① d10–d24 DCI group was slightly higher than that in ② d14–d24 DCI group, although the difference was not significant (surface markers: *P* = 0.695; cytoplasmic markers: *P* = 0.604). The ① d10–d24 DCI protocol was therefore used for subsequent experiments. Immunofluorescence labelling of differentiated iPSCs also revealed the presence of a small number of CCSP^pos^/SPC^pos^ cells (Fig. 2a).

### Ultrastructure of differentiated iPSCs

To analyse the ultrastructure of iPSC-derived BASCs, the sorted Sca-1^pos^/CD45.2^neg^/CD31^neg^ cells were examined using TEM. Club cells have been shown to contain a large number of secretory granules [48], and the presence of lamellar bodies and microvilli has been identified as a characteristic of both normal-lung AT-2 cells and ESC/iPSC-derived AT-2 cells [35, 39, 49]. Here, the sorted cells containing secretory granules harboured microvilli, as well as small and immature lamellar body-like structures. The secretory granules varied in number among the cells, and the lamellar body-like structures were concentrated in the vesicles (Fig. 2b).

### Intratracheal transplantation of differentiated iPSCs into naphthalene-treated mice

**Fig. 3:**
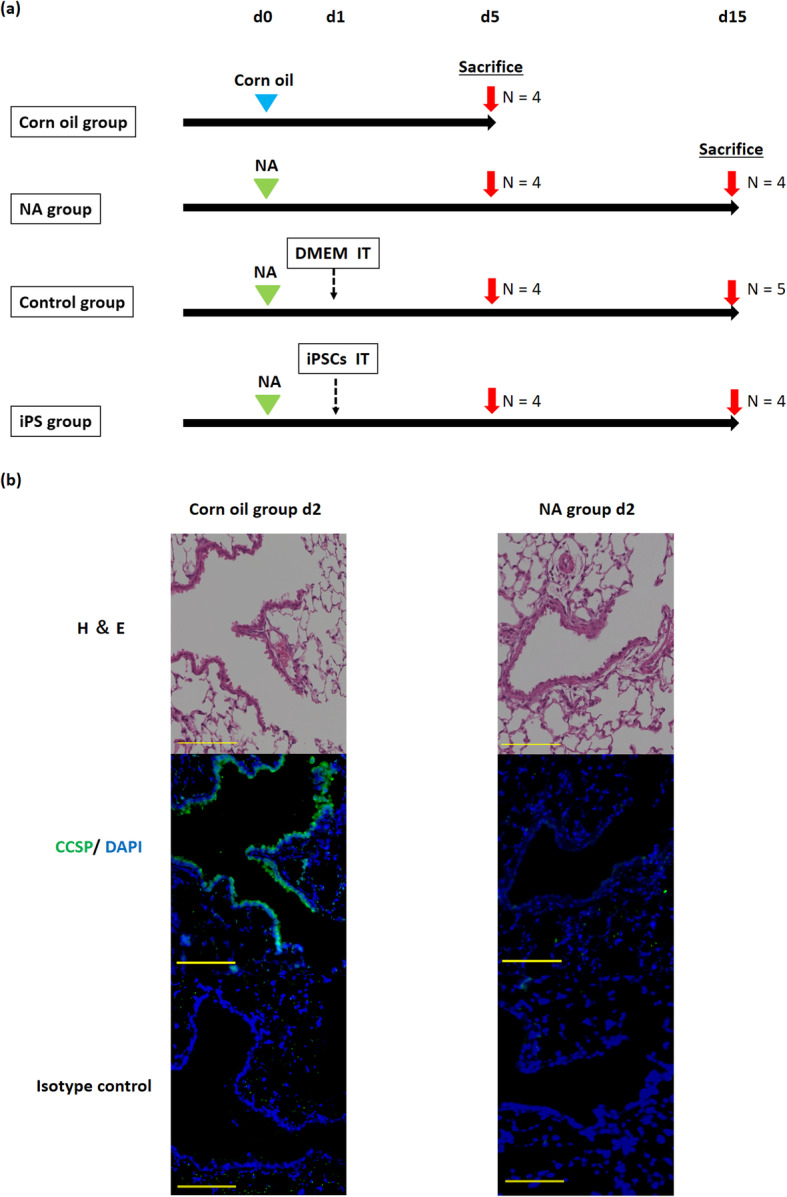
Establishment of mouse distal airway injury model. **a** Schematic showing experimental design. Corn oil or naphthalene was injected intraperitoneally into mice at 0 days. At 1 days, DMEM and differentiated iPSCs were intratracheally administered in the control and iPS groups, respectively. Mice were sacrificed at 5 days (*n* = 4 for all groups) and 15 days (*n* = 4 for NA group and iPS group, *n* = 5 for control group). **b** Preparation of naphthalene-treatment model. Corn oil and NA groups are shown from the second day after treatment. In the NA group, CCSP-positive club cells (green) were completely detached. NA, naphthalene treatment; iPSCs, induced pluripotent stem cells; IT, intratracheal injection; BW, body weight; H&E, haematoxylin and eosin; CCSP, club cell secretory protein; DAPI, 4′,6-diamidino-2-phenylindole dihydrochloride

To obtain a mouse distal airway injury model, naphthalene was intraperitoneally administered to female mice (Fig. 3a). Naphthalene, a club cell-specific cytotoxicant, was reported to cause particularly strong damage in female mice [11, 12, 50]. In this injury model, club cells were almost completely depleted on the second day after naphthalene treatment; however, the cells were normally retained after the administration of corn oil (Fig. 3b).

**Fig. 4:**
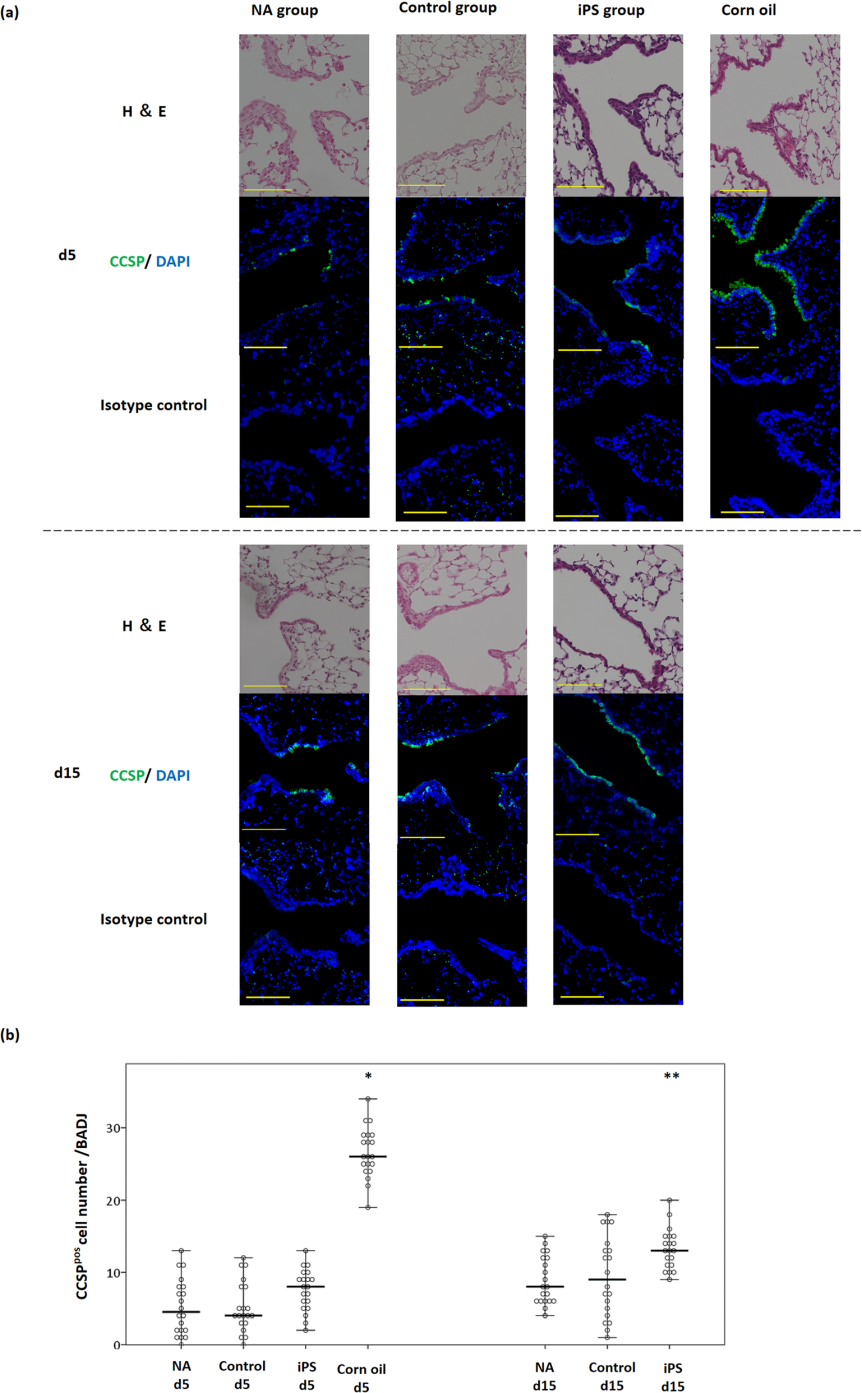
Quantification of CCSP-positive club cells at BADJs on day 5 and 15 after naphthalene treatment. **a** Immunofluorescence labelling of club cell marker CCSP (green) in each group. Nuclei were counterstained with DAPI (blue). Scale bar = 100 μm. **b** Dot-and-whisker plot showing numbers of CCSP-positive cells at BADJs. Horizontal line represents median and whiskers show min and max values. *P < 0.01, **P < 0.05 vs all other groups; one-way ANOVA followed by Tukey’s multiple-comparison test. CCSP, club cell secretory protein; BADJs, bronchioalveolar-duct junctions; DAPI, 4′,6-diamidino-2-phenylindole dihydrochloride

CCSP-positive cells at BADJs were then labelled and quantified after different treatments (Fig. 4a). At 5 days, the mean numbers of these cells in the NA, control, iPS, and corn oil groups were 5.3 ± 3.9, 5.2 ± 3.5, 8.1 ± 3.3, and 26.6 ± 3.5, respectively. The number of CCSP-positive cells was significantly increased in the corn oil group (*P* < 0.001) but did not differ between the other groups (iPS group vs control group, *P* = 0.198; iPS group vs NA group, *P* = 0.216). At 15 days, the mean numbers of CCSP-positive cells at BADJs in the NA, control, and iPS groups were 8.9 ± 3.3, 9.5 ± 5.6, and 13.3 ± 2.8, respectively. The recovery of CCSP-positive club cells was significantly promoted in the iPS group (iPS group vs control group, *P* = 0.013; iPS group vs NA group, *P* = 0.004) (Fig. 4b).

### Engraftment of differentiated iPSCs

**Fig. 5:**
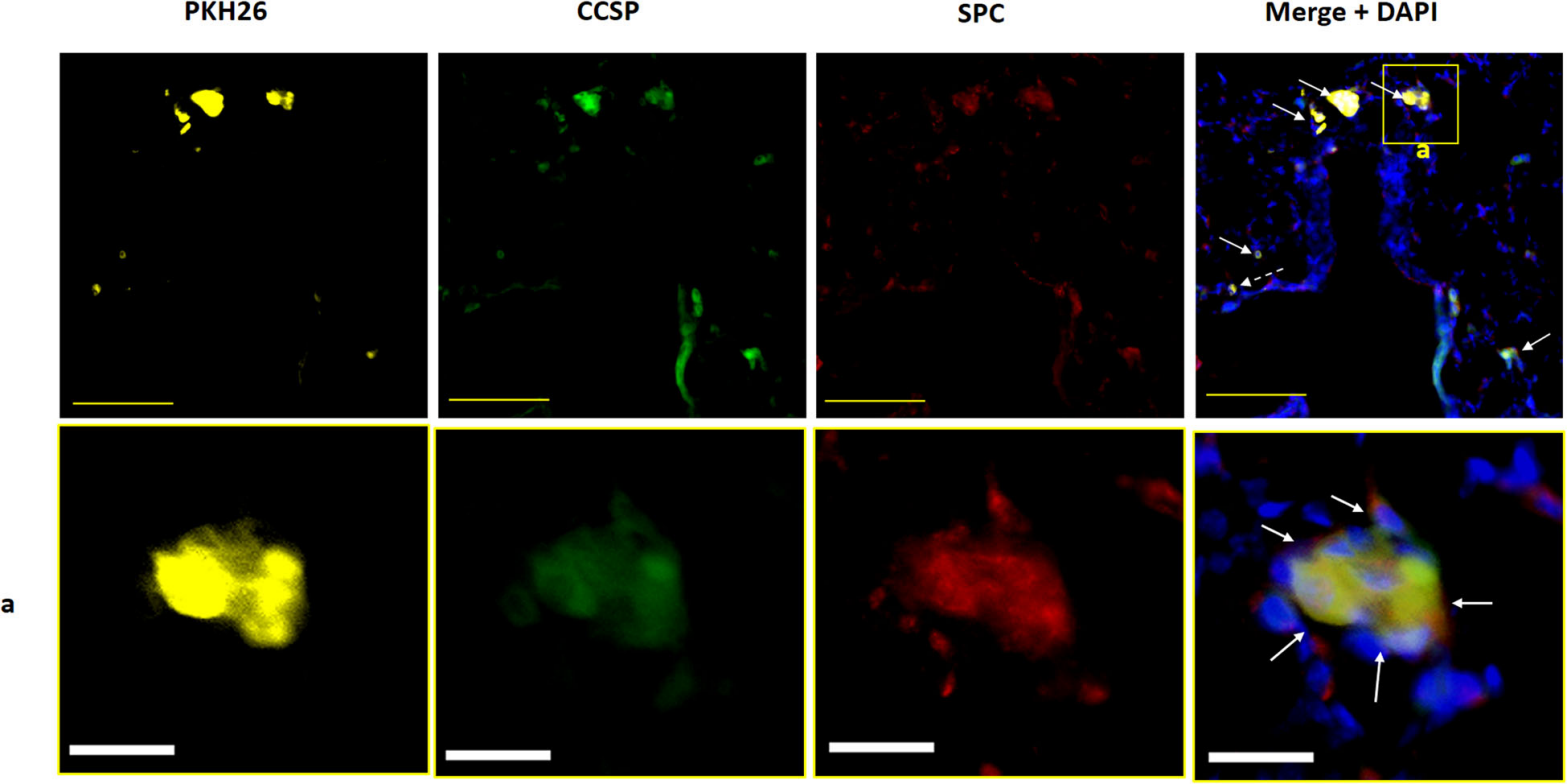
CCSP and SPC immunofluorescence in lung sections 5 days after intratracheal transplantation of differentiated iPSCs. Differentiated iPSCs were labelled with PKH26 cell tracker (yellow). Nuclei were counterstained with DAPI (blue). PKH26-positive, CCSP-positive (green), and SPC-positive (red) cells (PKH^pos^/CCSP^pos^/SPC^pos^ cells, white arrow), which are BASCs, were present around BADJs. PKH^pos^/CCSP^neg^/SPC^neg^ cells (white dotted arrow) were also detected. Bottom panels: magnified view of area **a** in top panel. PKH^pos^/CCSP^pos^/SPC^pos^ cells formed clumps. Yellow scale bar = 100 μm; white scale bar = 20 μm. CCSP, club cell secretory protein; SPC, surfactant protein C; iPSCs, induced pluripotent stem cells; BADJs, bronchioalveolar-duct junctions; DAPI, 4′,6-diamidino-2-phenylindole dihydrochloride

**Fig. 6:**
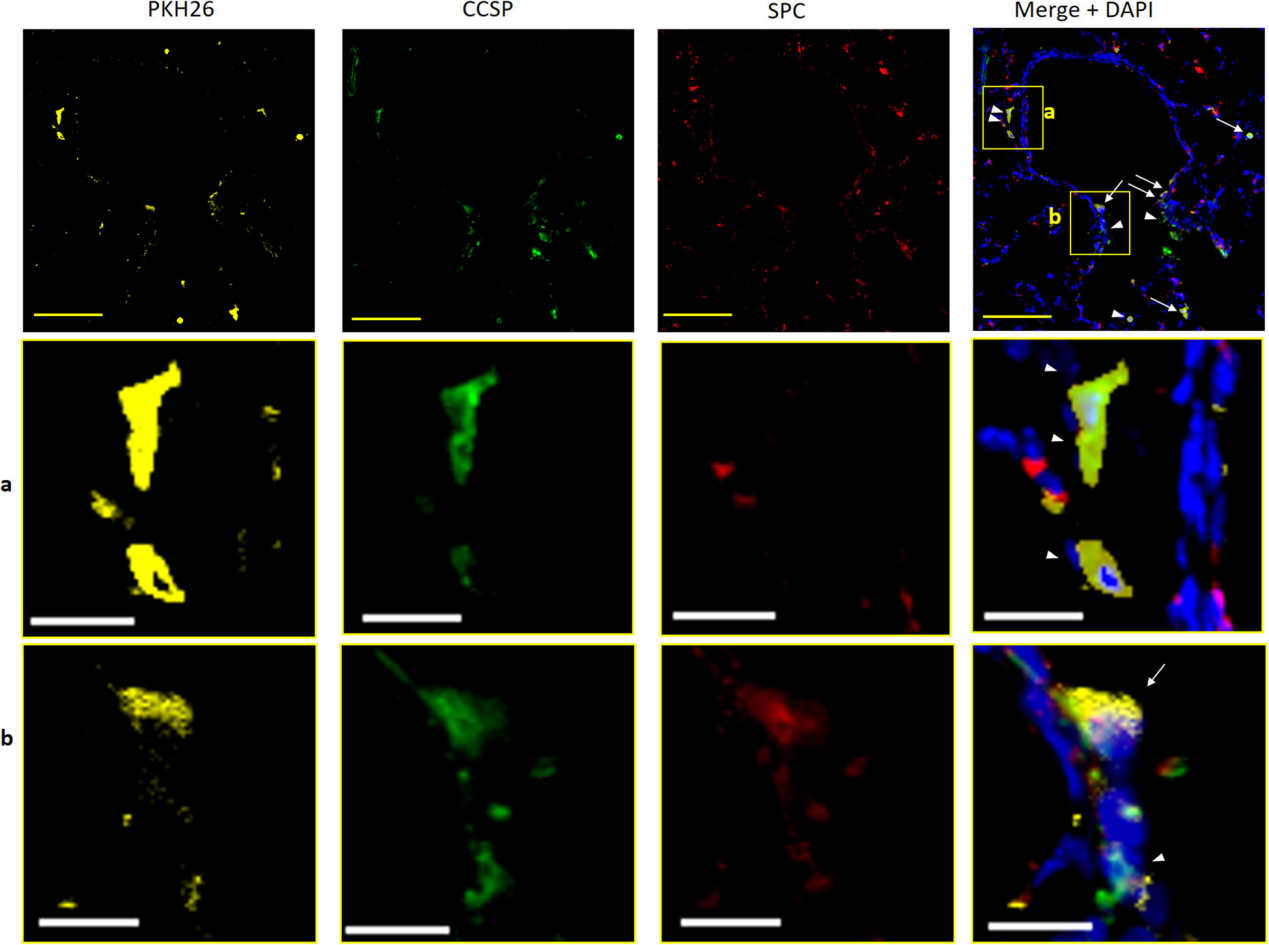
CCSP and SPC immunofluorescence in lung sections 15 days after intratracheal transplantation of differentiated iPSCs. Differentiated iPSCs were labelled with PKH26 cell tracker (yellow). Nuclei were counterstained with DAPI (blue). PKH26-positive, CCSP-positive (green), and SPC-positive (red) cells (PKH^pos^/CCSP^pos^/SPC^pos^ cells, white arrow), which are BASCs, were found as part of the epithelium of the BADJ. PKH^pos^/CCSP^pos^/SPC^neg^ cells (white arrowhead), which are club cells, were scattered. Middle and bottom panels: magnified view of areas **a** and **b** in top panel, respectively. CCSP, club cell secretory protein; SPC, surfactant protein C; iPSCs, induced pluripotent stem cells; BADJs, bronchioalveolar-duct junctions; DAPI, 4′,6-diamidino-2-phenylindole dihydrochloride

In frozen mouse lung sections, numerous PKH26-positive cells were detected in alveoli and terminal bronchioles at both 5 and 15 days, which verified the engraftment of transplanted differentiated iPSCs. The results of immunofluorescence labelling further demonstrated that these cells were PKH^pos^/CCSP^pos^/SPC^pos^ and PKH^pos^/CCSP^neg^/SPC^neg^, which confirmed the engraftment of the transplanted BASCs in mice at 5 days (Fig. 5). PKH^pos^/CCSP^pos^/SPC^pos^ and PKH^pos^/CCSP^pos^/SPC^neg^ cells were also similarly detected in mice at 15 d. Our results confirmed that the transplanted BASCs retained their stem cell characteristics for 2 weeks after transplantation and that the engrafted PKH^pos^/CCSP^pos^/SPC^neg^ cells constituted the BADJ epithelium (Fig. 6).

## Discussion

In this study, iPSC-derived BASCs transplanted into naphthalene-treated mice were shown to be engrafted in the lung, where they retained their BASC phenotype and promoted recovery from injury. Moreover, the ultrastructure of the iPSC-derived BASCs was revealed.

Naphthalene treatment in mice causes the selective shedding of terminal bronchiolar club cells; however, this effect is reversible, and recovery occurs spontaneously [12, 13]. Although the steady-state number of BASCs is very low, BASC numbers increase temporarily after naphthalene treatment and subsequently return to pre-treatment levels; therefore, BASCs are considered to play critical roles as local stem cells during the repair of club cells [6, 19, 22]. We hypothesised that transplantation of exogenous BASCs, which would serve to supplement the endogenous BASCs already present, might result in reduced damage and promote repair. In adult mice, BASCs account for < 1% of the total lung cells, and thus a comparatively richer BASC population must be obtained for cell transplantation [14, 29]. Here, iPSCs were successfully differentiated into BASCs.

Herein, to differentiate iPSCs into BASCs, the protocol developed by Schmeckebier et al. for differentiating iPSCs into AT-2 cells was adopted [39]. To date, no study has reported ESC or iPSC differentiation into BASCs. However, since BASCs are recognised as progenitor cells of AT-2 cells, it was postulated that BASCs could be obtained during the AT-2-cell differentiation process. The forementioned protocol was selected for the current study as it was reported to yield cells in which CCSP mRNA levels were higher than in an AT-2 cell line [35, 39]. In the study by Schmeckebier et al., the mRNA levels of both CCSP and SPC were high in ① d10–d24 DCI and ② d14–d24 DCI in the presence of KGF [39]. Therefore, the BASC-induction efficiency was compared in the ① d10–d24 DCI and ② d14–d24 DCI groups; however, no statistically significant differences were observed.

When BASCs were identified based on surface markers, Sca-1^pos^/CD45.2^neg^/CD31^neg^ cells were found to constitute 15.1 ± 7.8% and 12.8 ± 7.9% of the cell populations in ① d10–d24 DCI and ② d14–d24 DCI groups, respectively. Sca-1^pos^/CD45.2^neg^/CD31^neg^ cells in the BADJ of mouse lung can self-renew over multiple passages and differentiate into cells of airway and alveolar epithelium [14]. However, it has been reported that lung fibroblastic progenitor cells are part of the Sca-1^pos^/CD45.2^neg^/CD31^neg^ cell population [51]. Meanwhile, it has also been shown that resident Sca-1^pos^/CD45.2^neg^/CD31^neg^ cells in the lung undergo extensive self-renewal, differentiate into endothelial cells and lung epithelial cells in vitro, and have therapeutic potential for lung injury [52]. Therefore, the Sca-1^pos^/CD45.2^neg^/CD31^neg^ cell population may contain BASCs as well as various other cell populations. Conversely, with identification based on cytoplasmic markers that define true BASCs, the CCSP^pos^/SPC^pos^ cell populations were found to constitute 7.0 ± 5.3% and 4.9 ± 3.6% in ① d10–d24 DCI and ② d14–d24 DCI groups. The ratio of the BASC identification rate by surface markers to the rate by cytoplasmic markers was 46% and 38% in ① d10–d24 DCI and ② d14–d24 DCI groups, respectively. In a study conducted using total lung cells of adult mice, immunofluorescence labelling revealed that 85% of the Sca-1^pos^/CD45.2^neg^/CD31^neg^ cell population co-expressed CCSP and SPC [14]. However, the percentage calculated in the current study was < 85%. This disparity in results might be due to the difference between cultured cells in vitro and total lung cells in vivo.

The CCSP^pos^/SPC^pos^ cell population, which represents BASCs, accounted for a small proportion of the cells (7.0 ± 5.3%) in the ① d10–d24 DCI group, and these cells were transplanted intratracheally. However, when the differentiated iPSCs were intratracheally transplanted into naphthalene-treated mice, a higher proportion of engrafted cells were PKH^pos^/CCSP^pos^/SPC^pos^ cells, while the number of PKH^pos^/CCSP^neg^/SPC^neg^ cells was low (Figs. 5 and 6). Thus, cells displaying the BASC phenotype appeared to show an increased likelihood of engraftment in naphthalene-treated mice. Moreover, this phenomenon was observed in mice not only at 5 days, but also at 15 days, at which point the transplanted BASCs were retained in the lung and the cells maintained the BASC phenotype (PKH^pos^/CCSP^pos/^SPC^pos^).

Herein, PKH26 dye was used for cell tracking. The lipophilic tails of PKH26 diffuse into the cell membrane, leaving the fluorogenic moiety exposed near the outer surface of the cell. The intensity of PKH26 stain decreases linearly with each cell division [53]. This dye is often used for stem cell tracking as it does not require genetic modification or affect the proliferative capacity of cells [35, 54, 55]. However, if a labelled cell gives rise to a large number of progeny, or if the labelled cells require long-term (≥ 100 days) observation, the progeny may not contain sufficient membrane dye to be recognised. Because these issues were not faced in the current study, this dye was deemed suitable for use.

To date, a select few cell therapy studies have been conducted using similar naphthalene-injury models [5, 7, 8]. For instance, naphthalene treatment has been used not only for developing a model of airway injury to investigate potential therapies, but also as a ‘preconditioning regimen’ to create an airway-specific niche for cell incorporation [7, 8]. When bone marrow cells or tissue stem cells were intratracheally transplanted into naphthalene-treated mice in previous studies, engraftment of CCSP-positive cells at the BADJ was observed [5, 8]. BASCs were the focus of the current study, the results of which demonstrated not only engraftment of the cells in the injury area, but also enhanced repair of the injury caused by naphthalene. These findings suggested that iPSC-derived BASCs could represent a favourable cell source in cell therapy.

At 5 days, the number of club cells was slightly higher in the iPS group than in the NA and control groups; however, the difference was not significant. Meanwhile, at 15 days, 13.3 ± 2.8, 8.9 ± 3.3, and 9.5 ± 5.6 club cells at BADJs were observed in the iPS, NA, and control groups, respectively, and markedly enhanced club cell repair was observed in the iPS group. At 15 days, engraftment of PKH26-positive BASCs (PKH^pos^/CCSP^pos^/SPC^pos^) and PKH26-positive club cells (PKH^pos^/CCSP^pos^/SPC^neg^) at the BADJ was observed. It remains unclear whether the engrafted BASCs transdifferentiated into club cells or whether the club cells contained in the transplanted cell population were engrafted. Considering that the administration of cell-derived exosomes was reported to promote the proliferation of endogenous BASCs [56], one possibility is that exosomes from the transplanted iPSC-derived cells acted on endogenous cells to promote repair. A potential alternative mechanism may involve a pathway associated with thrombospondin-1 and the receptor CD47. Although the *OSKM* gene has been used to induce iPSCs, upregulation of this gene in impaired cells has been shown to promote repair. Alternatively, thrombospondin-1 secretion is induced by cell stress, which suppresses the expression of OSKM via CD47 [57, 58]. Hence, iPSC-derived cells may act on this pathway to promote repair. Moreover, the pathway by which paracrine fibroblast growth factor 10 from parabronchial smooth muscle activates BASCs was reported to be critical for epithelial repair after naphthalene-induced injury [59]. Furthermore, genetic-lineage tracking of BASCs in vivo showed that not all of the repaired airway epithelium was derived from BASCs [22]. Therefore, the transplanted cell population containing BASCs might engage in crosstalk with endogenous factors and cells and promote recovery from naphthalene-induced injury through direct cell repair or indirect effects.

Despite limitations associated with iPSC-derived cells, such as altered ultrastructure, here, the ultrastructure of BASCs was revealed for the first time. Although BASCs have been studied from various genetic perspectives, their ultrastructure had not been clarified [6, 14, 22, 23]. In the current study, iPSC-derived BASCs were found to harbour immature lamellar body-like structures and microvilli characteristic of AT-2 cells, as well as secretory granules, which are characteristic of club cells. This finding is in accord with the CCSP^pos^/SPC^pos^ phenotype of BASCs revealed by immunostaining.

To our knowledge, this study has shown for the first time that BASCs can be induced from mouse iPSCs. Moreover, this study confirmed not only that the iPSC-derived BASCs were engrafted in the mouse model of naphthalene-induced airway injury, but also that BASCs promoted repair, indicating that these cells can potentially be used for cell therapy. Although rapid progress has been made in deriving the lung epithelial lineage from ESCs and iPSCs, as reflected herein, a major challenge that remains is the generation of complex 3D tissue structures, or functional organs, from these cells. The functional structures that have been previously obtained by culturing cells in a monolayer, such as the retina or myocardial sheets, have been readily used in clinical applications [60, 61]. Recently, the regeneration of functional lungs has been attempted using decellularised lungs as a 3D scaffold followed by re-cellularisation [62, 63]. However, it has not yet been possible to replace the organ itself and maintain the function for an extended period. Moreover, the methodology required for delivering and retaining lung stem cell-based regenerative therapies in the injured lung remains under development. The transplantation of iPSC-derived progenitor cells, stromal cells isolated from human bone marrow and adipose tissue, and mesenchymal stromal cells to treat terminal-bronchiole/alveolar-region disorders (e.g. chronic obstructive pulmonary disease, bronchiolitis obliterans, acute lung injury) is expected to attenuate injury or promote regeneration [5, 35, 45–47, 64–66]. The findings obtained using our cell therapy method involving BASCs will also contribute to further development of treatments for terminal-bronchiole/alveolar-region disorders.

Certain limitations were noted in this study. First, all iPSC-derived differentiated cells were intratracheally transplanted, and BASCs, representing only 7.0 ± 5.3% of the cells, were not clearly shown to contribute to the enhanced repair. Since the number of BASCs required for transplantation cannot be readily obtained given the current induction efficiency, it will be necessary to further improve this efficiency and isolate BASCs for transplantation. However, the induction efficiency of AT-2 cells was reported to be 9–18%, and thus, it might be challenging to markedly increase the induction efficiency of BASCs [31, 35, 36]. In addition, investigation using a 3D organoid culture is needed to support the conclusion that iPSCs can be differentiated into BASCs and engrafted in the BADJ. Second, the observation period after cell transplantation was only 2 weeks. Hence, the long-term dynamics of the engrafted BASCs remain unknown. Thus, it will be necessary to devise a cell tracking method and perform long-term BASC tracking.

## Conclusions

Herein, mouse iPSCs were shown to be differentiated in vitro into cells exhibiting the BASC phenotype. Notably, the differentiated iPSCs promoted club cell repair at the BADJ in the mouse model of naphthalene-induced airway epithelium injury.

## Data Availability

Not applicable.

## Abbreviations

ANOVA: Analysis of variance
AT: Alveolar type
BADJ: Bronchioalveolar-duct junction
BASCs: Bronchioalveolar stem cells
BM: Basal differentiation medium
CCSP: Club cell secretory protein
DAPI: 4′,6-Diamidino-2-phenylindole dihydrochloride
DMEM: Dulbecco’s modified Eagle’s medium
EB: Embryoid body
ESCs: Embryonic stem cells
FACS: Fluorescence-activated cell sorting
H&E: Haematoxylin and eosin
iPSCs: Induced pluripotent stem cells
KGF: Keratinocyte growth factor
OCT: Optimal cutting temperature
SPC: Surfactant protein C
TEM: Transmission electron microscopy
